# Impact of human body shape on free convection heat transfer

**DOI:** 10.1371/journal.pone.0318842

**Published:** 2025-02-06

**Authors:** Shri H. Viswanathan, Ankit Joshi, Lyle Bartels, Kambiz Sadeghi, Jennifer K. Vanos, Konrad Rykaczewski

**Affiliations:** 1 School for Engineering of Matter, Transport and Energy, Arizona State University, Tempe, AZ, United States of America; 2 Julie Ann Wrigley Global Futures Laboratory, Arizona State University, Tempe, AZ, United States of America; 3 School of Sustainability, Arizona State University, Tempe, AZ, United States of America; Memorial Sloan Kettering Cancer Center, UNITED STATES OF AMERICA

## Abstract

Understanding the thermal comfort and safety of diverse populations within indoor settings requires a quantitative understanding of the primary heat exchange pathways between occupants and their surroundings: radiation and free convection. Thus far, however, free convective heat transfer coefficients have only been determined for the average Western adult. To this end, we investigated how variation in body shape impacts free convection heat transfer using an experimentally validated numerical model. The multiphysics model was compared against experiments conducted using the thermal manikin ANDI ("Advanced Newton Dynamic Instrument") in a climate-controlled enclosure across five air-to-skin temperature differences ranging from 4.9 to 13.9°C. The difference between measured and simulated heat fluxes for the whole body, and per anatomical region, was typically <5%, occasionally reaching 15–20%, for some body regions due to physical features not modeled in the virtual ANDI model. Using the validated model, we simulated free convection around a family, or diverse group, of virtual manikins representing the 1^st^ to 99^th^ percentile body mass index (BMI) and height variation in the United States adult population. Our results show that the free convection heat transfer coefficient is independent of human sex and height but decreases slightly with increased BMI. However, the variation from the average manikin in the whole body and regional free convection coefficients with BMI was small, not exceeding 8% and 16%, respectively. Furthermore, our regression coefficients and exponents can be derived from the theorical correlation for free turbulent convection from a vertical plate, which also explains the observed independence of the heat transfer coefficient from the manikins’ height. Overall, these findings demonstrate the general applicability of using an average body shape in indoor thermal audits and/or overheating risk assessments to understand thermal comfort and heat stress. The results and valid application of the model support critical insights for human health, productivity, and well-being connected to heat and cooling in buildings.

## 1. Introduction

Industrialization and urbanization have resulted in people in the United States (U.S.) spending, on average, over 90% of their time in indoor settings [1]. According to the American Society of Heating, Refrigerating and Air-Conditioning Engineers (ASHRAE), the acceptable operative temperatures for thermal comfort in such spaces range from 20.5°C to 27°C with the air speed not exceeding 0.2 m·s^-1^ [2, 3] However, with the increasing frequency and intensity of heat waves [4], indoor settings can often be substantially warmer than these recommendations, especially in buildings that lack or do not use air conditioning due to economic or emergency reasons [5, 6]. If an indoor environment is too warm, it can negatively impact the occupants’ health and productivity [7–11]. Accordingly, there is an increased focus on research [12] and policy [13] related to indoor overheating. To predict whether a specific warm environment will lead to heat stress on an occupant, a quantitative understanding of the dominant heat exchange pathways in indoor settings—radiation and free convection (that is driven by air buoyancy and is dominant when air speed below 0.2 m·s^-1^) [2, 3, 14]—between a human body and the surroundings is required.

Methods to quantify dry heat exchange between the human body and indoor surroundings have evolved significantly over the last 80 years. In early studies, the convective and radiative heat transfer coefficients were measured using human subjects through direct and partitional calorimetry methods [15–17]. These human trials were tedious to conduct, and the results were affected by intra- and inter-subject physiological variabilities [18, 19]. Subsequently, researchers began using physical or computational representations of the human body ranging in geometrical complexity from a single or series of cylinders to anatomically representative multizone thermal manikins [20–27]. Several studies have utilized these instruments to understand the effect of postures and motion on free convective heat transfer coefficients [21, 23, 25, 27–33]. While thermal manikins typically represent an average Western male or female (50^th^ percentile body mass index (BMI) and height), they fail to capture the diversity in body morphology across broader populations. Apart from one study focusing on neonatal babies [34], research in the past has not quantitatively examined how variations in body shape due to BMI and height influence free convective heat transfer in populations beyond the Western "average". This represents a significant knowledge gap, particularly given that obesity is associated with increased risks of heat illness [35, 36]. Therefore, understanding body shape’s role in free convection is important for developing inclusive strategies to mitigate indoor overheating risks in diverse populations [35, 36].

Here, we address this knowledge gap by employing an experimentally validated numerical model to determine the variation of the whole-body and regional free convection coefficients across adult body shapes representing the diversity of the adult U.S. population. Specifically, we developed a coupled convection and radiation simulation that is experimentally validated within a controlled thermal environment with a physical thermal manikin ANDI (i.e., Advanced Newton Dynamic Instrument). For the validation, the coupled radiation and convection numerical model replicates the three-dimensional geometry of the experimental chamber and the manikin. After experimental validation, we applied the simulations to determine free convection coefficients for the computational manikin "family" representing the 1^st^ to 99^th^ percentile BMI and height variation in the United States adult population [37, 38]. Hence, we provide a deeper understanding of the influence of shape and body characteristics on convective and radiative exchanges, resulting in more accurate representation in models applied within indoor thermal audits and/or overheating risk assessments.

## 2. Methods

### 2.1. Sealed enclosure and climatic chamber

**Fig 1:**
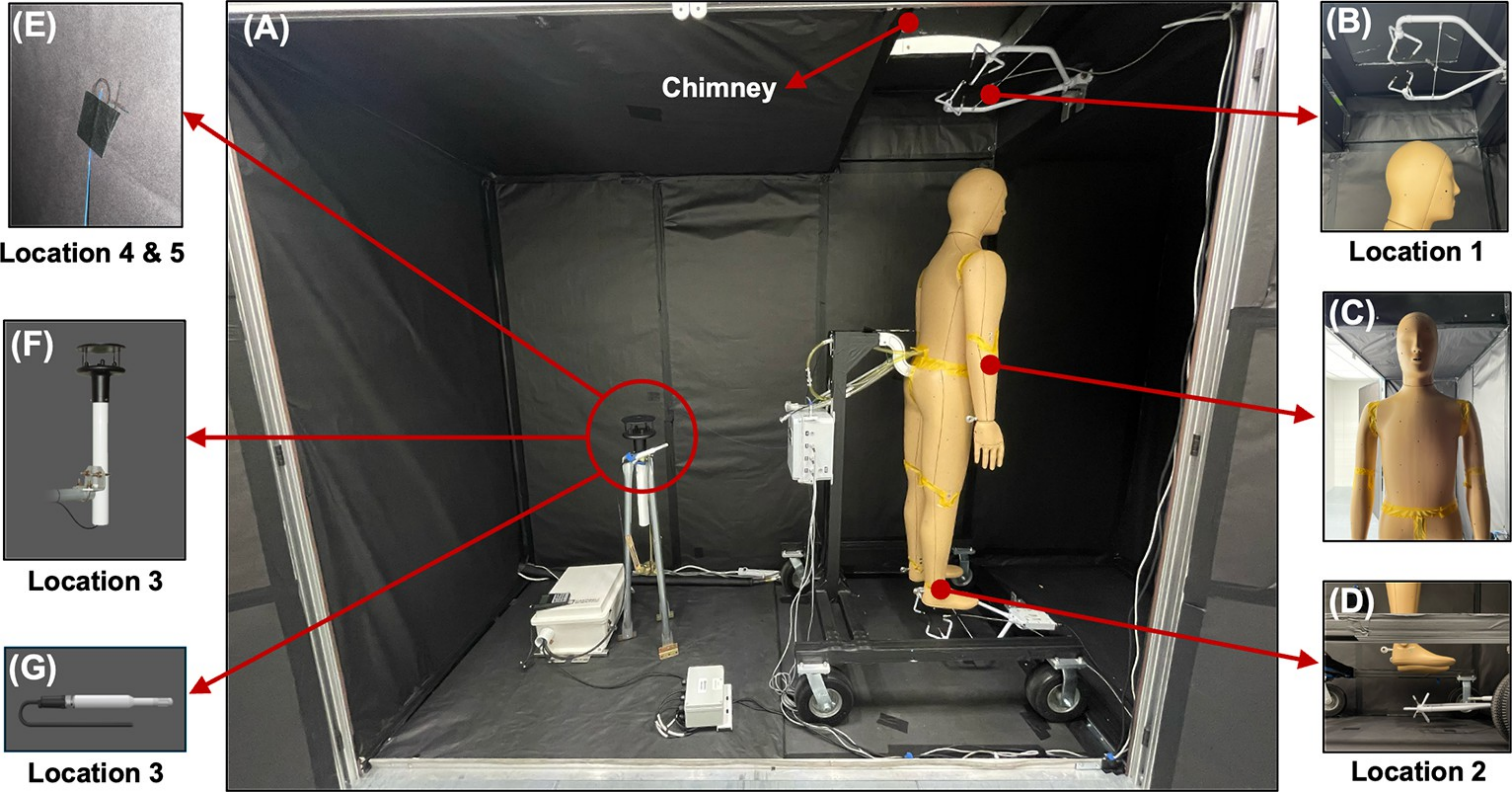
Experimental setup and probe locations inside the sealed enclosure. (A) Thermal manikin ANDI inside the sealed enclosure, (B)-(G) Locations of probes described in Table 1 and **Table SM 1**.

We conducted experiments within a sealed controlled enclosure (2.28 m height, 1.14 m width, 3.5 m length) located inside a climatic chamber (3 m height, 2.75 m width, and 6 m length) [39] as shown in Fig 1A (see S1A, S1B Fig in S1 File). The climatic chamber’s temperature and relative humidity can be controlled independently. The sealed enclosure was built using 15 mm aluminum T-slot extrusions and polycarbonate panels (Faztek Industrial Solutions). Since these panels can reflect longwave radiation under certain angles, we covered all the inner walls of the enclosure, chimney, and floor with highly emissive black paper (Duo-Finish Kraft Paper Roll–1.2 m by 66 m from Pacon Corporation) to simplify the computational modeling process. The longwave spectroscopic properties of the black paper were measured using a PerkinElmer Frontier Fourier Transform Infrared Spectrometer (FTIR) [40, 41], with the longwave emissivity of this paper being 0.99. To purge the rising hot plume from the enclosure, we added a chimney (0.31 m height, 1.14 m width, and 0.81 m length) with an outlet (0.5 m x 0.5 m), as shown in Fig 1B.

### 2.2. Instrumentation

We used a customized thermal manikin, "Advanced Newton Dynamic Instrument" (ANDI), manufactured by Thermetrics LLC, Seattle, USA. ANDI represents the 50^th^ percentile western male with 35 independently controllable body zones [40]. The manikin stands 1.78 m tall with a total body surface area of 1.86 m^2^ (see S2 Fig in S1 File illustrating the cross-sectional schematic of ANDI’s shell). Each zone has surface-distributed resistive temperature sensors that are collected and communicated to the ThermDAC software at 5 second intervals. The manikin was placed inside the controlled enclosure below the chimney’s outlet, as illustrated in Fig 1B. While conducting the controlled experiments, we observed that a small fraction of the rising hot air plume tends to enter the hollow manikin shell through its actuated joints. This phenomenon increases the whole-body total heat flux by 3% to 6.3% (see S3 Fig in S1 File). Hence, ANDI’s joints (armpits, elbows, waist, crotch, knees, and ankles) were covered with masking tape, as depicted in Fig 1C. ANDI was attached to a non-reflective mobile stand from the lower back, causing the manikin to lean forward approximately by 8.3° with its feet 0.25 m above the ground, as shown in Fig 1D. While the manikin has fingers, they are not heated and are not considered during experiments and analysis. The ambient temperature and relative humidity are measured using an EE181 probe manufactured by Campbell Scientific, which collects data every 3 seconds. The placement and description of the additional sensors used during experimentation are summarized in Fig 1A–1G, Table 1, and S1 Table in S1 File [29, 30]. Though both anemometers provide directional information, only the resolved magnitude of the air speed was analyzed. The exterior surface of the manikin had a long wave emissivity of 0.98 [40, 41].

**Table 1:** *Locations corresponding to Fig 1A–1G and specifications of the air and wall temperature and air flow probes used to characterize the enclosure environment*. We note that the manufacturer specified the uncertainties of the 3D sonic anemometer in absolute terms and the 2D sonic wind sensor in relative terms.

Probe	Variable	Quantity	Uncertainty	Frequency (Hz)	Location
3D Sonic Anemometer–CSAT3B	Air speed	2	± 0.08 m·s^-1^ (U_x_, U_y_) ± 0.04 m·s^-1^ (U_z_)	1	1, 2
2D Sonic Wind Sensor–WINDSONIC1-L	Air speed	1	± 2%	1	3
EE181	Air temperature	1	± 0.2°C	0.33	3
T-type Thermocouple	Wall temperature	2	± 0.5°C	1	4, 5

### 2.3. Experimental procedure

To measure the whole-body and localized total dry heat exchange between the manikin and the enclosure, we conducted controlled experiments with five constant air temperature (T_air_) levels ranging from 29.1 to 20.1°C measured within the sealed enclosure (see Table 2). The experiment was repeated thrice for each air temperature, resulting in 15 experiments. To prevent water concentration impacts on air density [42], the absolute humidity within the enclosure was always maintained constant at 9.28 g of water per kg of dry air. Before starting the experiment, we ensured the enclosure was sealed entirely, such that the air within the enclosure was nearly stationary (air speed < 0.02 m·s^-1^). Once the air within the enclosure became stable, the manikin was switched on. The thermal manikin can be operated in either the constant surface temperature or heat flux mode. We utilized the constant temperature mode because it closely replicates the human skin temperature in a thermally neutral environment [25]. Additionally, conducting experiments in constant temperature mode avoids the complications of accounting for radiative exchange between different sections of the manikin, which may be at different temperatures in the constant heat flux mode. Nevertheless, as discussed in Section 3.1, the computationally derived free convective heat transfer coefficients were consistent across both modes. We set the surface temperature (T_surf_) of all 35 manikin surface zones to 34°C, which is typically the mean skin temperature [14]. The difference between the manikin T_surf_ and the surrounding T_air_ was computed and given as ΔT_surf-air_. Each experiment lasted 75 minutes, with manikin surface temperature changes in the final 30 minutes being within 0.05°C (and therefore utilized in calculations). The thermal environment recorded for each condition inside the enclosure is tabulated in Table 2. We note that the air temperature within the sealed enclosure (T_air_) was 1°C to 2°C higher than that measured outside the enclosure but still within the climatic chamber (T_chamber_). Air temperature within the enclosure was used in the analysis. The manikin measurements were recorded for all zonal parameters every five seconds. The total whole-body heat flux was computed using segmental area weighting of the zonal time-averaged data. This process was followed for all the repetitions at each T_air_ setting.

**Table 2:** *Experimental conditions for the five experimental runs with the manikin within the controlled enclosure*.

Run	Chamber air temperature (°C)	Chamber relative humidity (%)	Enclosure air speed (m·s^-1^)	Manikin surface temperature (T_surf_) (°C)	Enclosure air temperature (T_air_) (°C)	Δ*T*_*surf*−*air*_ (°C)
1	28	37.2	0.01	34	29.1 ± 0.2	4.9 ± 0.2
2	25	44.3	0.01	34	26.4 ± 0.2	7.6 ± 0.2
3	23	50.0	0.02	34	24.5 ± 0.2	9.5 ± 0.2
4	21	56.3	0.01	34	22.7 ± 0.2	11.3 ± 0.2
5	18	67.7	0.01	34	20.1 ± 0.2	13.9 ± 0.2

The Type A, or experimental error (*u*_*A*_), and the Type B, or instrumental error (*u*_*B*_), for each zonal heat flux were calculated individually and utilized to determine the combined standard uncertainty (*u*_*C*_). We multiplied the combined standard uncertainty with a coverage factor (k) to expand the uncertainty. For the two-tailed Student’s t-Distribution with 2 degrees of freedom (3 repetitions) and 90% confidence level, the coverage factor is 2.92.


$$u_{C}=\sqrt{u_{A}^{2}+u_{B}^{2}}$$ Eq 1

$$U=ku_{C}$$ Eq 2

### 2.4. Coupled radiative, flow, and heat transfer simulation

**Fig 2:**
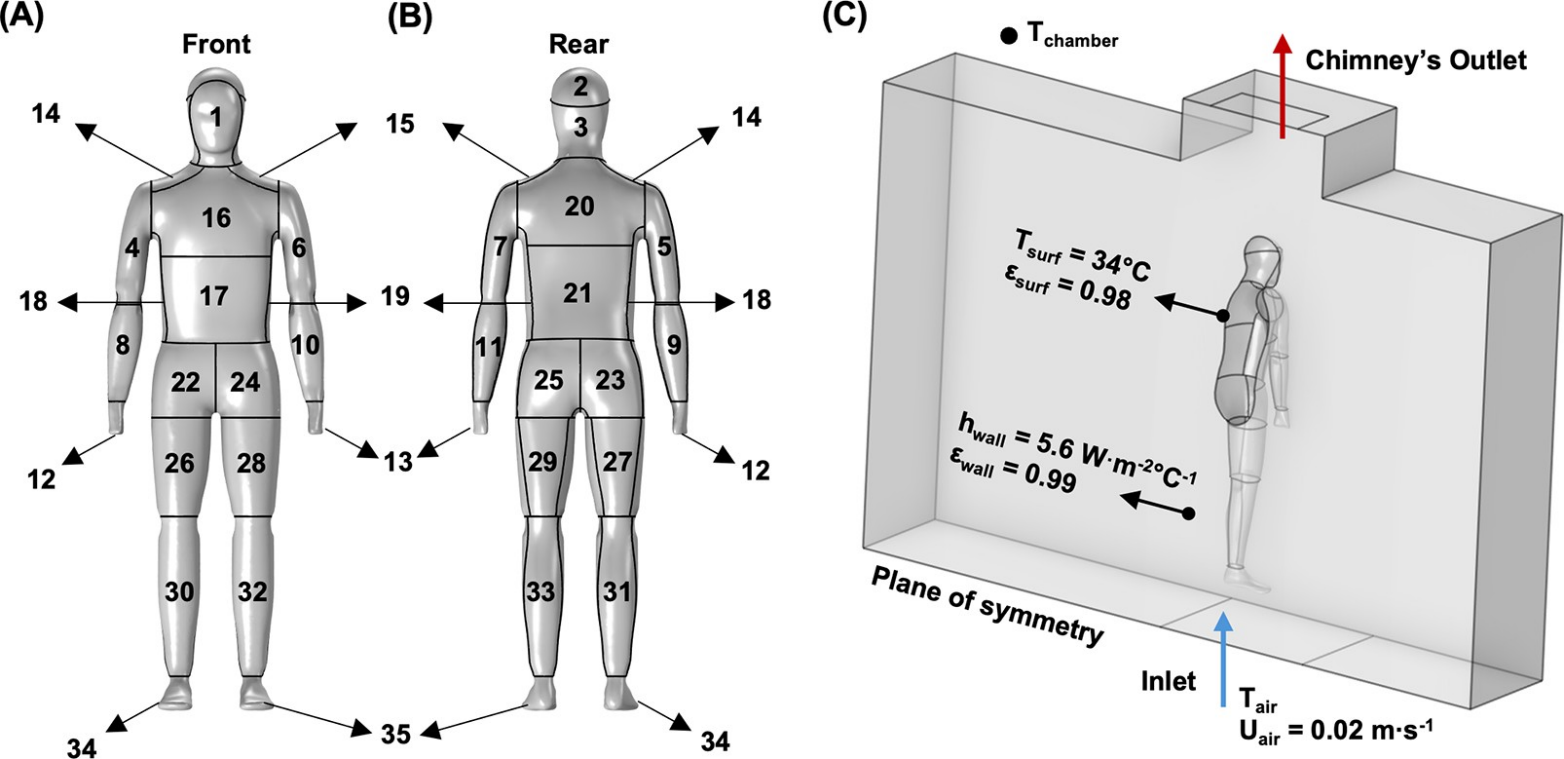
Schematic of ANDI’s segments and coupled simulation model with key boundary conditions. (A) Front and (B) Back view schematic showing ANDI’s 35 segments and (C) Schematic of the coupled simulation model, where T_surf_−manikin surface temperature (°C), ε_surf_−emissivity of the manikin surface, h_wall_–equivalent heat transfer coefficient (W·m^-2^°C^-1^) of the walls encompassing wall resistance and external convection, ε_wall_–emissivity of the wall, T_air_–enclosure air temperature (°C), U_air_–enclosure air speed (m·s^-1^), and Tchamber−chamber air temperature (°C).


We simulated the coupled air flow, radiation, and convection around the manikin using finite element method-based software COMSOL Multiphysics 6.1. The computer model of ANDI’s zonal shells was imported into the Autodesk Meshmixer software to edit and clean 3D surface mesh. All the individual body zones (3D surface mesh) were combined using plane cut and bridge functions. The bridged edges were subsequently smoothened using the "robust smooth" function. ANDI’s refined watertight surface mesh was exported as a.stl file into the COMSOL Multiphysics software. The mesh was segmented into 35 individual body zones at the surface level using plane cut and adapt functions, as illustrated in Fig 2A and 2B. The whole-body surface area of the virtual ANDI was 1.7% greater than that of the physical ANDI. However, some virtually segmented zonal areas differed moderately from the actual zonal areas (see S2 Table in S1 File for zonal discrepancies). Following the segmentation, the mesh was imported into the COMSOL geometry node. After the geometry of the controlled enclosure was created, the orientation and location of the virtual ANDI within the enclosure were adjusted to match the experimental setup, as shown in Fig 2C. Symmetry conditions were applied along the sagittal/median plane to reduce the computational time. Since the physical manikin does not have heated fingers, these features were excluded from the computational model.

ρ∇∙u=0 Eq 3

$$\rho (\frac{\partial u}{\partial t})+\rho (u∙\nabla u)=\nabla ∙[-pI+K]+F+\rho g$$ Eq 4

$$K=(\mu +\mu_{T})(\nabla u+{\left(\nabla u\right)}^{T})$$ Eq 5

$$\mu_{T}=\rho C_{\mu }k^{2}f_{\mu }/\varepsilon$$ Eq 6

$$P_{k}=\mu_{T}[\nabla u:(\nabla u+{\left(\nabla u\right)}^{T})]$$ Eq 7

$$\rho (\frac{\partial k}{\partial t})+\rho (u∙\nabla )k=\nabla ∙\left[(\mu +\frac{\mu_{T}}{\sigma_{k}})\nabla k\right]+P_{k}-\rho \varepsilon$$ Eq 8

$$\rho (\frac{\partial \varepsilon }{\partial t})+\rho (u∙\nabla )\varepsilon =\nabla ∙\left[(\mu +\frac{\mu_{T}}{\sigma_{\varepsilon }})\nabla \varepsilon \right]+{P_{k}C}_{\varepsilon 1}\varepsilon /k-\rho C_{\varepsilon 2}\varepsilon^{2}f_{\varepsilon }/k$$ Eq 9

$$\rho C_{p}(u∙\nabla T)+\nabla ∙(k\nabla T)=Q+Q_{P}+Q_{vd}$$ Eq 10

The governing equations for the model are presented in **Eqs 3 & 4** (Continuity Equations), **5–9** (Turbulent Kinetic Energy (k) and Dissipation Rate (ε) Transport Equations) & **10** (Heat Transfer), where *u* is the velocity flow field [m·s^-1^], *ρ*–density [kg·m^-3^], *F*–External forces [N], *g*–acceleration due to gravity [9.81 m·s^-2^], *μ*–dynamic viscosity [m^2^·s^-1^],], *μ*_*T*_–Turbulent viscosity [m^2^·s^-1^], *σ*_*k*_–Turbulent Prandtl Number for k, *σ*_*ε*_–Turbulent Prandtl Number for ε, *P*_*k*_–production rate of turbulent kinetic energy [kg·m^-1^·s^-3^], *p*–pressure [Pa], *C*_*ε*1_, *C*_*ε*2_–Empirical constant, *T*–temperature [K], *k*–thermal conductivity [W·(m·K)^-1^] and *Q* –internal heat sources [W·m^-3^]. The simulation coupled the "Turbulent flow", "Heat transfer in solids and fluids", and "Surface-to-surface radiation" modules available within the software. We used the Reynolds Averaged Navier Stokes (RANS) method to solve the fluid flow physics employing Low Re k-ε turbulence model with Low Re wall functions. This approach is well-suited to resolving flow behavior in low-turbulence regions, enabling precise modeling of the bulk flow and boundary layer without requiring excessive mesh refinement near the walls [43]. To facilitate numerical convergence, we simulated an air inlet at the bottom of the enclosure with a negligible air speed of 0.02 m·s ^-1^ (with 5% turbulent intensity and geometry-based turbulence length scale), which did not impact the simulation outcomes. In addition, to avoid convergence issues related to steady-state solution with transition from laminar to turbulent regions, we simulated a 10 second transient period during which a quasi-steady state with under 0.5% heat transfer coefficient variation was achieved after 3 seconds. Since air was considered an incompressible medium, we used the Boussinesq approximation [43].

As in the experiments, the manikin surface temperature was set to 34°C. The inlet air temperature was set to the experimental temperature measured at location 3, as shown in Fig 1G, and positioned 1.2 m behind the manikin at its waistline height (1 m). During experiments, the temperature of the climatic chamber surrounding the sealed enclosure was 1–2°C cooler than the T_air_ measured at position 3. Based on empirical matching of the wall temperatures experimentally measured at locations 4 and 5 across five T_air_ settings, we applied an effective heat transfer coefficient of 5.6 W·m^-2^°C^-1^ on the walls of the enclosure (see S4A–S4B Fig in S1 File). Fluid flow and heat exchange physics were coupled using the "Non-Isothermal Flow" multiphysics module. Finally, to solve the radiosity equations, surface-to-surface radiation (hemicube method with resolution of 64) was used [37, 44]. Since all the surfaces were assumed to be diffuse and gray, the surface radiative properties were set to "wavelength independent". "Heat transfer in solids and fluids" and "surface-to-surface radiation" modules were also coupled using the multiphysics module "Heat transfer with surface-to-surface radiation".

Two cuboidal refinement volumes were created surrounding the manikin surface to create high-density mesh elements. The floor of the enclosure was meshed using free triangular distribution. Except for the manikin surface and the refinement zones, the rest of the enclosure was swept with prism mesh elements. We created boundary layers at the manikin surface to compute velocity and temperature gradients closer to the surface. Following the COMSOL Multiphysics software’s recommendation [43], we maintained the "distance to the cell center in viscous units" equal to or below 0.5 by adjusting the number of the boundary layers to 15, stretching factor to 1.2, and the total thickness of the boundary layers to 2 cm. We conducted a mesh refinement study to determine when the free convective heat transfer coefficient becomes independent of the number of elements (see S5A, S5B Fig in S1 File). The final mesh consists of 360100 elements with a maximum element size of 7.2 cm, a minimum element size of 1.6 cm, an element growth rate of 1.14, a curvature factor of 0.5, and a resolution of narrow region of 0.8. The average element quality is 0.71. Post-meshing, we solved this numerical model using a time-dependent study with wall distance initialization and a duration of 10 seconds (see S6A, S6B Fig in S1 File).

Once free convective (C_Sim_) and radiative (R_Sim_) heat fluxes were determined computationally, we separated the measured total heat fluxes (Q_T_) by subtracting the simulated radiative heat flux from it, leaving behind the experimental free convective heat flux (C_Exp_) (see **Eq 11)**. We note that the radiative component of the simulation was previously validated against Fanger’s experimental results [37, 44]. Finally, we divided the free convective heat fluxes by the temperature difference (ΔT_surf-air_) to obtain the free convective heat transfer coefficients (h_WB.Exp_ and h_WB.Sim_), as shown in **Eqs 12** and **13**.


$$C_{Exp}{=Q}_{T}-R_{Sim}$$ Eq 11

$$h_{WB.Exp}=C_{Exp}/\Delta T_{surf-air}$$ Eq 12

$$h_{WB.Sim}=C_{Sim}/\Delta T_{surf-air}$$ Eq 13

The results were fitted to a power function using the NonLinearModelFit function in Wolfram Mathematica 13.2 with weights of one over uncertainty square and 90% confidence interval [45].

### 2.5. The virtual manikin family with varied body shapes

**Fig 3:**
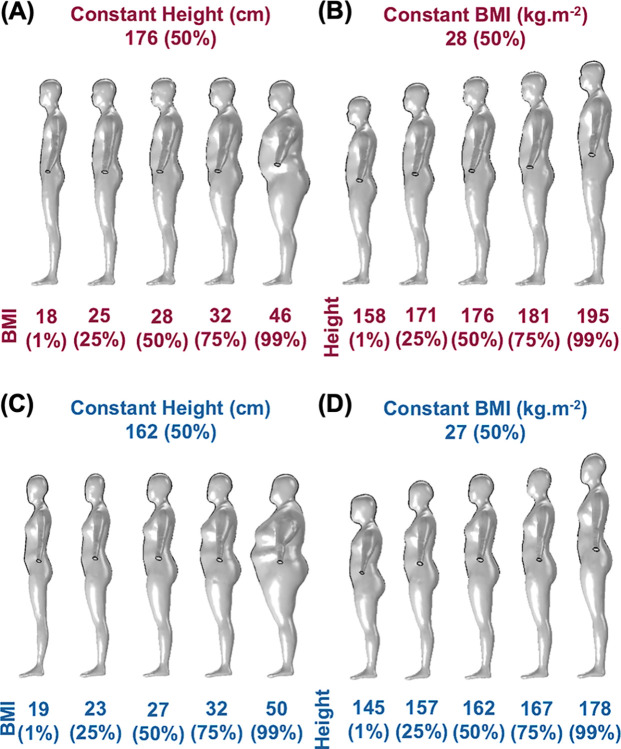
Family of virtual manikins with varied body shapes. (A) Male manikins with constant height (50^th^ percentile) but varying BMI (1^st^–99^th^ percentile), (B) Male manikins with constant BMI (50^th^ percentile) but varying height (1^st^–99^th^ percentile), (C) Female manikins with constant height (50^th^ percentile) but varying BMI (1^st^–99^th^ percentile) and (D) Female manikins with constant BMI (50^th^ percentile) but varying height (1^st^–99^th^ percentile).

We simulated free convection around the "virtual manikins" family representing the adult population of the U.S. We used BMI (kg·m^-2^) and height (cm) to describe the body shape of the "virtual manikins". We generated these virtual manikins by extracting the raw 3D human body models from the Open Design Lab Manikin Fetcher tool based on the United States National Health and Nutrition Examination Survey (NHANES) [46]. As comprehensively described in our previous works [37, 44], these models were cleaned and processed to generate watertight body meshes. To gain insight into the impact of the two body characteristics on convection, initially, we simulated 9 cases each for male and female manikins: one set with a constant BMI (50^th^ percentile) and varying height (1^st^–99^th^ percentile variation) and another with a constant height (50^th^ percentile) and varying BMI (1^st^–99^th^ percentile variation) (see Fig 3A–3D). Additionally, 4 extreme cases were considered for each sex, including combinations of 1% height–1% BMI, 1% height–99% BMI, 99% height–1% BMI, and 99% height–99% BMI, resulting in a total of 26 simulations. These manikins were imported into the convective numerical model and placed at the geometric center of a virtual room (2.5 m height, 2 m width, and 3 m length; see S7A, S7B Fig in S1 File). Though the virtual room geometrically differs from the sealed enclosure described earlier, we note that the enclosure geometry does not impact the whole-body free convective heat transfer coefficients (h_WB_) (see Results Section 3.1). The boundary conditions for the convective model were identical to those described in Section 2.4. Since the virtual room and the manikin geometries are symmetrical, we simulated one-half of the geometry to reduce the computational run time. A mesh refinement study for the virtual room was also performed (see S5C Fig in S1 File), and the final mesh settings were a maximum element size of 6.52 cm, minimum element size of 1.23 cm, element growth rate of 1.13, curvature factor of 0.5, and a resolution of narrow region of 0.8.

## 3. Results

### 3.1. Experimental validation of the coupled radiation and convection model using thermal manikin

**Fig 4:**
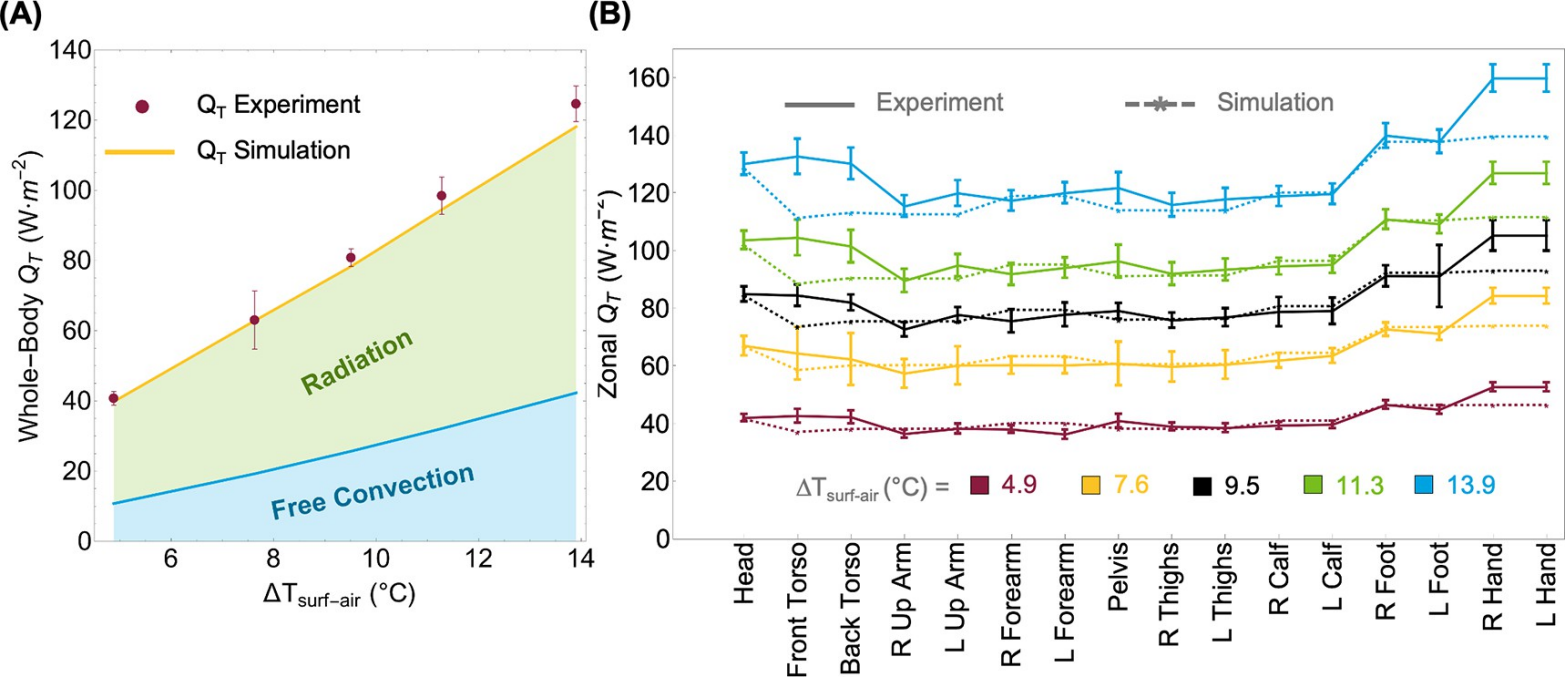
Comparison of whole-body and regional heat fluxes between experiments and simulations for various temperature differences. (A) Whole-body and (B) anatomical region total heat flux (Q_T_) measured through the experiments against the numerical simulation at five different temperature differences ($\Delta T_{surf-air}=T_{surf}-T_{air}$).

Fig 4A and 4B compares the whole-body and regional total heat flux obtained using experiments and simulations for five temperature differences (ΔT_surf-air_) ranging from 4.9°C to 13.9°C. In whole-body terms, there is close agreement between the numerical model and the manikin experiments, with the former underpredicting the experiments only by 0.4 to 4% for ΔT_surf-air_ up to 11.3°C, which is within the measurement uncertainties. At the highest ΔT_surf-air_ of 13.9°C, the numerical model underpredicts the experimental value by 5.2%, slightly outside the experimental uncertainty. To facilitate comparison of local experiments and simulations, we grouped ANDI’s 35 zones into 16 larger anatomical regions typically reported for convection measurements [40] through area-weighted summation of the original zones (see S3 Table and S8A, S8B Fig in S1 File).

Fig 4B shows good experimental and numerical heat flux agreement for most anatomical regions and temperature settings, with discrepancies over the experimental uncertainty (5% or so) consistently emerging for a few regions and being exacerbated at higher temperature differences. The simulated heat fluxes from the head, pelvis, lower limbs, forearms, and upper arms agree well with the experimental values (discrepancies lower than 5%). At the highest temperature difference, simulations of heat flux from the pelvis and left upper arm demonstrate a slightly higher discrepancy (up to 6.3%). We observed higher experimental to numerical disagreement for hands (11.5–12.6%), front (8.9–16%), and back torso (3.4–13%), with a maximum difference of 16% observed at the front torso at the highest temperature difference. The variations in the simulated and measured values for the hands likely stem from the physical ANDI only having a heated palmar region with "dummy" fingers that are not heated. Instead of fingers, the virtual ANDI has extruded palms that are geometrically different from the physical ANDI’s palms. In the simulation, the entire extruded palm was heated, whereas in experiments, only the front and rear surfaces of the palms were heated.

**Fig 5:**
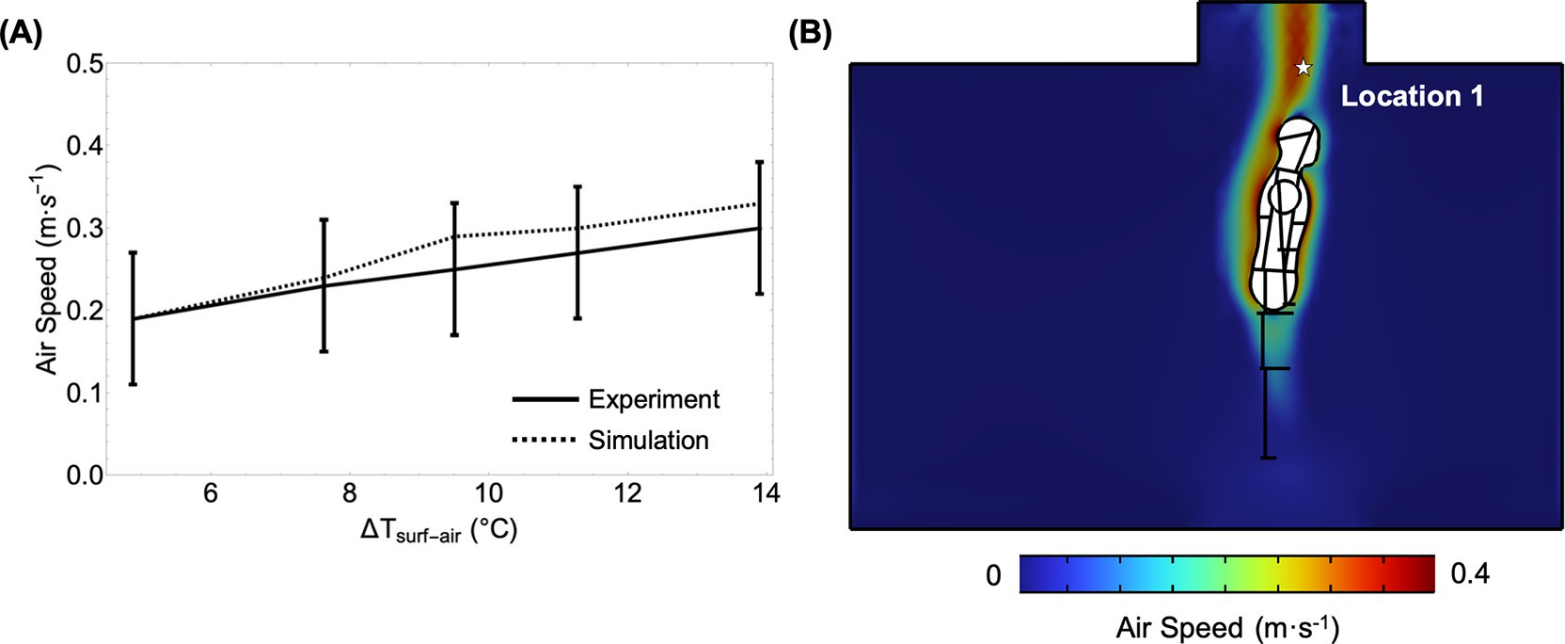
Characteristics of air speed around the thermal manikin. (A) Comparison of air speed measured at location 1 (illustrated in Fig 1B) through experiments against numerical simulation at five different temperature differences ($\Delta T_{surf-air}=T_{surf}-T_{air}$) and (B) Spatial distribution of the air speed of the rising plume around the manikin is illustrated.

To gain insight into the largest discrepancy observed in the torso region, we can evaluate it in terms of the spatially denser ANDI zones that comprise the torso anatomical region. However, no trend in heat flux related to height or front/back location of these zones emerges, with underprediction of the experimental heat fluxes for the chest (6–12.4%) and lower back (5.4–15.3%) being consistently smaller than for the stomach (12.5–18.9%) and the shoulders (12.1–19.3%). The most likely reasons for the numerical underprediction of the lower back heat flux are the presence of the stand attachment and the connecting cables and tubing that are not modeled computationally (compare the image in Fig 1A to the schematic in Fig 2C). In addition, on the physical manikin, these zones are surrounded by large, taped-over joints that are not modeled. In any case, agreement between the experiments and the numerical model ranges from excellent for most zones and tested temperature differences to, at worst, reasonable (~15 to 20%) for the torso under the largest temperature difference. Considering that a scatter of theory and experiments of 20% is expected for most engineering convection correlations [47], the experiments validate our numerical simulation. Additionally, the air speed of the rising plume measured at location 1 (illustrated in Fig 1B) agreed well with the spatially corresponding computational air speed, further validating our numerical simulation (see Fig 5A and 5B).

**Fig 6:**
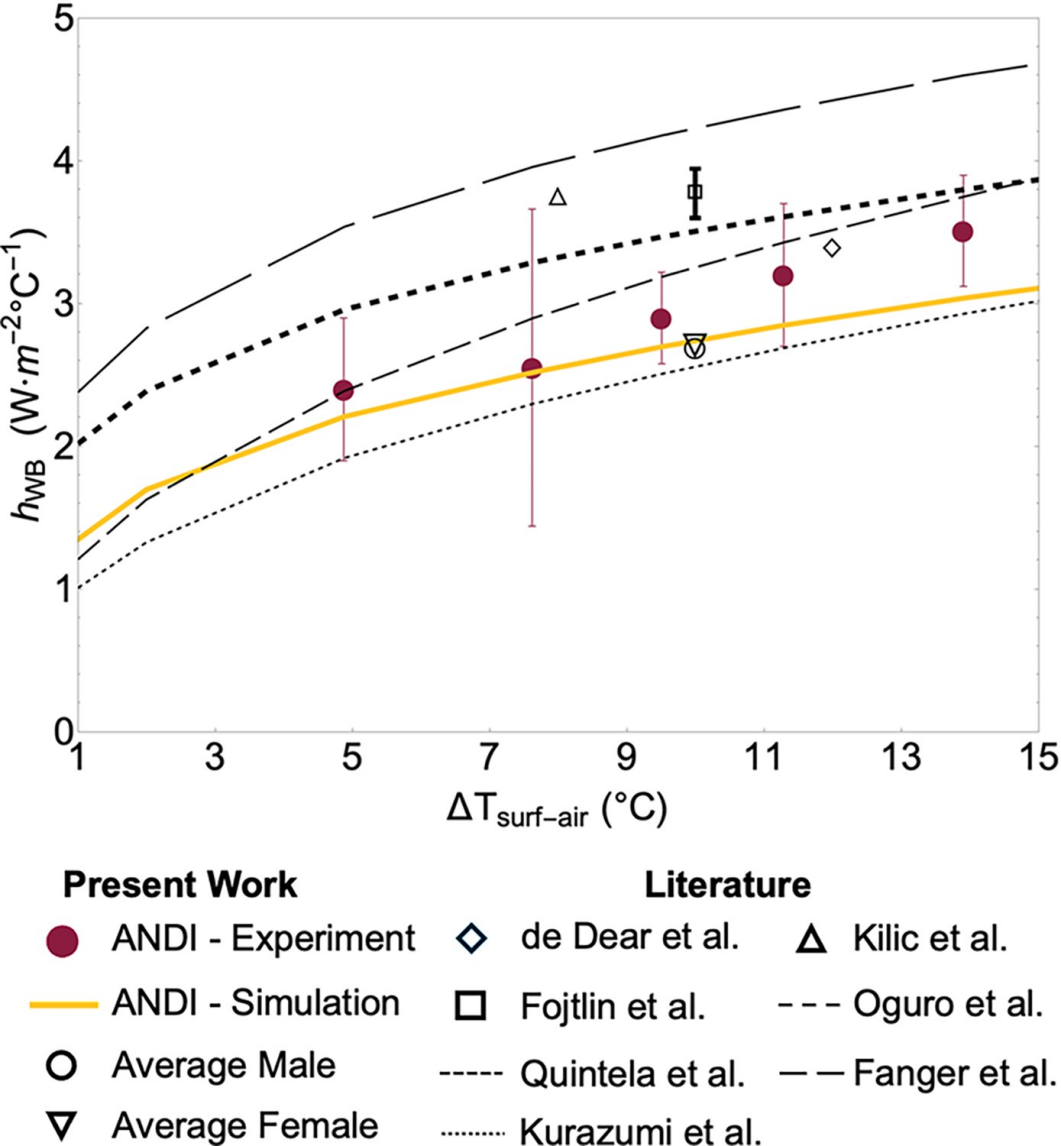
Comparison of whole-body free convective heat transfer coefficients (h_WB_) from experiments and simulations and with literature results (values from Fanger [20], de Dear et al. [21], Kilic et al. [22], Kurazumi et al. [23], Quintela et al. [24], Fojtlín et al. [25], and Oguro et al. [26] are shown). Our simulation results for average male and female manikins with ΔT_surf-air_ of 10°C from Fig 3 are also shown.

The computational and experimental whole-body free convection coefficients (h_WB_) were calculated by subtracting the computational radiative flux (see the green-shaded region in Fig 4A) from the respective total heat fluxes and dividing the remainder by ΔT_surf-air_. The computational radiative results had been previously validated against Fanger’s results [23]. Fig 6 shows the variation of the experimental (h_WB Exp_) and computational (h_WB Sim_) whole-body heat transfer coefficients with ΔT_surf-air_, along with results from prior literature. The different conditions corresponding to the literature data are summarized in S4 Table in S1 File. In a few cases, coefficients for only a single temperature difference were reported in the literature, while other groups provided regressions with the generic form of $h_{WB.Sim}={A(\Delta T_{surf-air})}^{B}$. We tabulate these prior correlations along with regressions to our measurements and simulations in Table 3 (regional equivalents are presented in the S5 Table in S1 File).

**Table 3:** Comparison between the coefficients and exponents of the nonlinear regression power function of $h_{WB.Sim}={A(\Delta T_{surf-air})}^{B}$ with 90% confidence interval values and with correlations from prior literature. The input temperature difference is in °C.

Reference	A	B
ANDI–Simulation	1.36±0.007	0.30±0.002
ANDI–Experiment	1.26±0.18	0.38±0.065
Quintela et al.	2.02	0.24
Oguro et al.	1.21	0.43
Kurazumi et al.	1.01	0.41
Fanger	2.38	0.25
Mean prior literature	1.65	0.33

As expected from the heat flux results, our h_WB_ values obtained using ANDI measurements and simulations agree well for most temperature differences. At worst, for ΔT_surf-air_ of 14°C, our simulation yields 2.93 W·m^-2^°C^-1^, which is slightly outside the experimental range of 3.51 ± 0.4 W·m^-2^°C^-1^ (95% confidence interval). This discrepancy in our data is small compared to the scatter between prior correlations for the average manikin from the literature [20–26], which consistently differ by up to 1.6 W·m^-2^°C^-1^. In contrast, for ΔT_surf-air_ of 10°C, our simulation of the whole-body heat transfer coefficient for the average male (2.69 W·m^-2^°C^-1^) and female (2.72 W·m^-2^°C^-1^) manikins (see Fig 3) within the virtual room (2.5 m tall, 2 m wide, and 3 m long) are essentially the same as simulations (2.72 W·m^-2^°C^-1^) and measurements (2.9 ± 0.32 W·m^-2^°C^-1^ for ΔT_surf-air_ of 9.5°C) for ANDI within the sealed enclosure. This observation also confirms that the enclosure or virtual room geometries do not influence the whole-body heat transfer coefficients. Importantly, this agreement demonstrates that we can use simulations within the virtual room to quantify the impact of various body shapes on natural convection. We also conducted a comparison simulation with a constant uniform heat flux across the manikin surface (80 W·m^-2^ or 1.38 MET) that yields an average surface temperature of about 10°C. The whole-body free convective coefficient for this uniform surface heat flux case (2.73 W·m^-2^°C^-1^) closely matched the h_WB Sim_ values for ΔT_surf-air_ of 10°C obtained for constant temperature across the manikin surface. This agreement indicates that the manikin operation mode has a negligible impact on the convection measurements; however, for experiments, we chose the constant temperature mode to facilitate implementation and analysis (i.e., avoiding considering manikin zone-to-zone radiative exchange).

### 3.2. Variation of free convection heat transfer with human body shape

**Fig 7:**
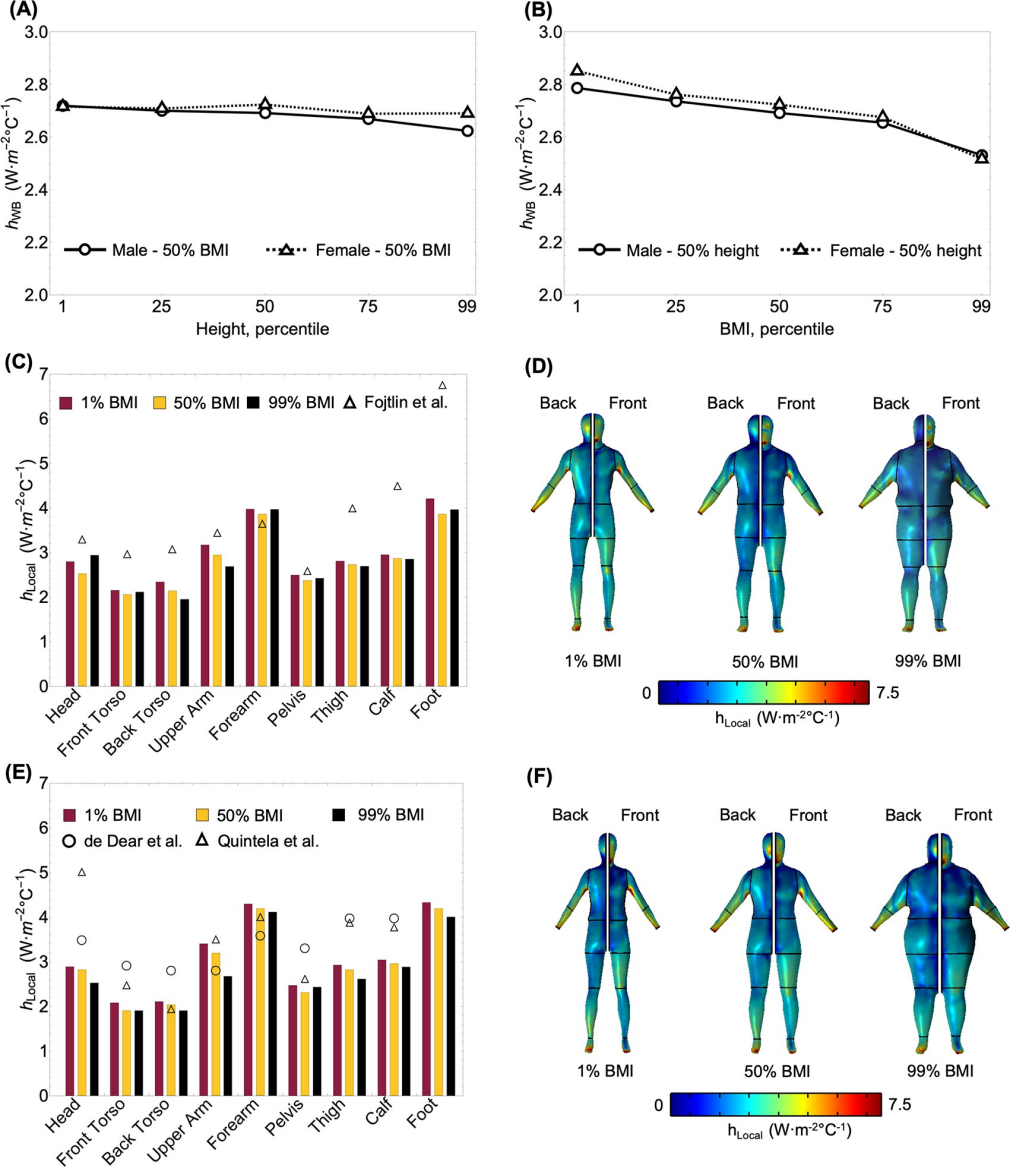
Effect of height and BMI on whole-body and local free convection coefficients for male and female manikins. (A) & (B) exhibit the effect of height and BMI on the whole-body free convection coefficient (h_WB_) for male and female manikins, respectively; (C) & (D) surface distribution and regional values of local free convective coefficients (h_Local_) among the male manikins with 50% height and 1%, 50%, 99% BMI and (E) & (F) surface distribution and regional values of local free convective coefficients (h_Local_) among the female manikins with 50% height and 1%, 50%, 99% BMI.

To quantify the effect of body shape on h_WB_, we simulated convection around each of the manikins shown in Fig 3A–3D within the virtual room with ΔT_surf-air_ of 10°C. Although there are substantial variations in the geometry of the manikins (see Fig 3), all the h_WB_ values lie within the narrow range of 2.52 and 2.85 W·m^-2^°C^-1^ (Fig 7A, 7B). The largest deviation from the average h_WB_ values were 2.53 W·m^-2^°C^-1^ (-6%) and 2.52 W·m^-2^°C^-1^ (-7.6%) for 50% height and 99% BMI female and male manikins, respectively. From Fig 7A, 7B, we can determine that this variation is driven by the change in the BMI of the manikins rather than their height or sex. In particular, changing the height from 1% to 99% while keeping the BMI at 50% leads to a negligible -1 to +2.5% deviation from the average h_WB_ for both male and female manikins. In contrast, an equivalent change in BMI with height constant at 50% leads to a more substantial -4.7 to +7.6% deviation from the average h_WB._ We also note that changing the geometrical body descriptors simultaneously (BMI and height) did not significantly impact the outcomes. For example, the h_WB_ value for the 99% BMI and 1% height female manikin is 2.54 W·m^-2^°C^-1^, which is the same as for the 99% BMI and 50% height female manikin. In other words, altering the height did not impact the whole-body free convection coefficient, yet a change in BMI did. To gain further insight into this change, we evaluate the impact of BMI on regional free convection coefficients.

Fig 7C–7F illustrates the free convection coefficient surface and regional distributions for average height male and female manikins with 1%, 50%, and 99% BMI. The bar plots (Fig 7C and 7E) show that the regional coefficient decreases or remains nearly constant with increasing BMI for most anatomical zones. The foot and forearm exhibit larger coefficients, with the highest being for the foot (h_foot_) at 4.21 W·m^-2^°C^-1^ for 1% BMI male and 4.33 W·m^-2^°C^-1^ for 1% BMI female manikins, which are 9% and 3.3% greater than h_foot_ of the average male and female manikin, respectively. In contrast, the torso region typically exhibits smaller values, with the lowest coefficient being for the back torso (h_back torso_) at 1.95 W·m^-2^°C^-1^ for 99% BMI male and 1.9 W·m^-2^°C^-1^ for 99% BMI female, which are 9% and 6.6% lesser than the h_back torso_ of average male and female manikins, respectively. However, variations in the whole-body (not exceeding 8% from the average) and regional (not exceeding 16% from the average) free convection coefficients with body shape are minor, especially as compared to the scatter in values reported in the literature for average manikins (see Figs 6 and 7C and 7E).

## 4. Discussion

### 4.1. Our free convection heat transfer coefficients in the context of theory and prior literature

The differences between our simulated and measured whole-body and regional free convection coefficients for ANDI and the differences related to change in body shape are minor compared to the scatter in values reported in prior literature for average body shapes [20–26]. For example, Kurazumi et al. [23] h_WB_ values were 3.7–12.9% lower than our h_WB.Sim_ values, while Oguro et al. [26] and Quintela et al. [24] values were 8.4–34.2% greater. A corresponding scatter translates to the regression coefficients and exponents, but our results are near the mean of all the prior correlations (see Table 3). In addition, our regressions can be directly related to classical free convection theory.

The near 1/3 exponent in our regression indicates the flow is transitioning to turbulent (vs. 1/4 for purely laminar flow) [47], which is expected from the Rayleigh number exceeding 10^9^ (i.e., $Ra=g\beta \Delta T_{surf-air}H^{3}/\nu \alpha$ ranges from 2 to 7*10^9^ for Δ*T*_*surf−air*_ of 5°C to 14°C with *g*–acceleration due to gravity [9.81 m·s^-2^], *β*–coefficient of volume expansion of air [1/T for ideal gases], H–ANDI’s height [1.78 m], *v*–air kinematic viscosity [m^2^·s^-1^], and *α*–air thermal diffusivity [m^2^·s^-1^]). In particular, for *Ra* between 10^9^ and 10^13^, the average heat transfer coefficient for free convection from a vertical plate can be calculated according to $h=0.1k_{air}{Ra}^{1/3}/H$ [47]. To cast in the same form as our regressions, we can re-arrange the vertical plate correlation to $h=0.1k_{air}{\left(g\beta /\nu \alpha \right)}^{1/3}{\Delta T}_{surf-air}^{1/3}$. Substituting property values, we obtain $h=1.18{\Delta T}_{surf-air}^{1/3}$. The 1.18 coefficient obtained from the vertical plate theory is just slightly lower than that from regressions to our ANDI experiments (1.26±0.18) and simulations (1.36±0.007). A slight enhancement in heat transfer for the human body can be expected since its geometry is more complex than that of a vertical plate. Therefore, our experimental and simulation results for the average male can be derived using classical theory for free convection from a vertical plate. This theory also explains the independence of the h_WB_ from the height of all the simulated manikins (see Fig 7A). Specifically, with the 1/3 exponent in the turbulent regime, the height of the manikin (or plate) is canceled out in the heat transfer coefficient calculation. Next, we discuss potential reasons for deviations of prior literature from our results.

**Fig 8:**
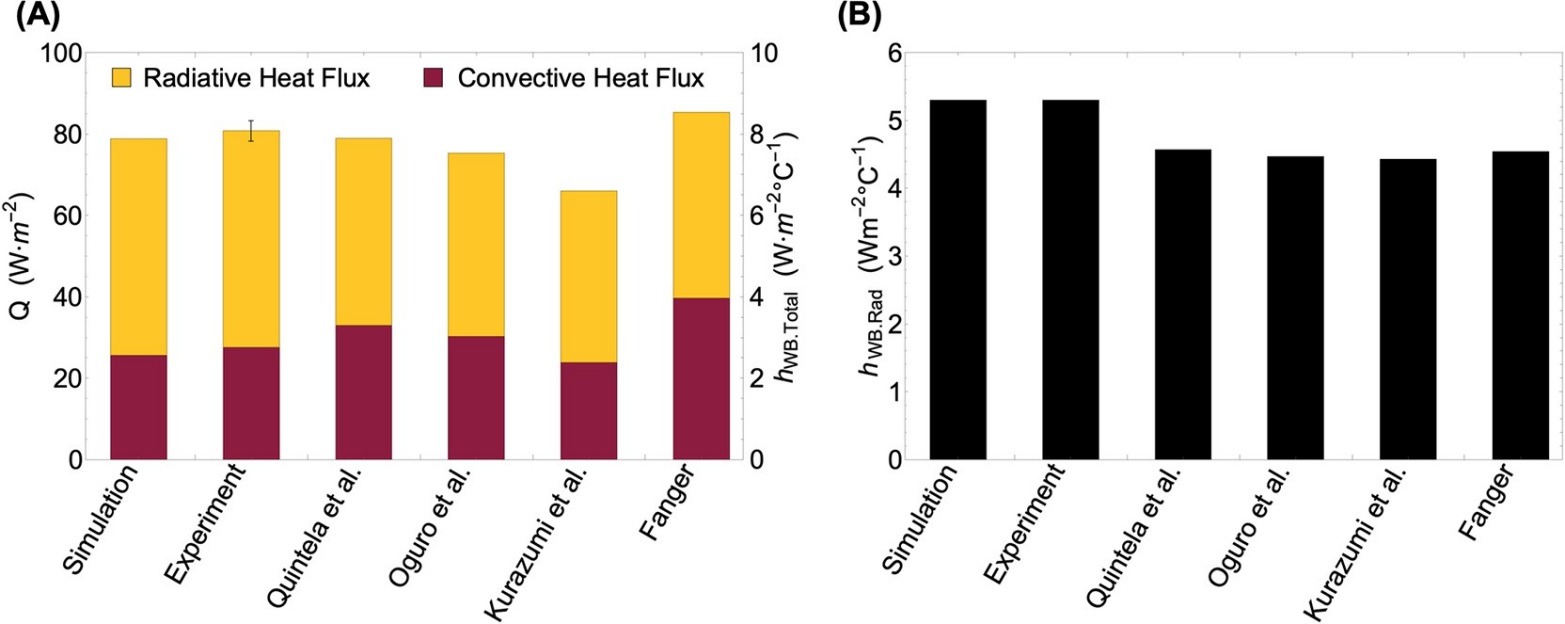
Comparison of heat fluxes and radiative coefficients with experiments, simulations, and literature. (A) Comparison of the whole-body total, radiative, and free convective heat fluxes and (B) radiative heat transfer coefficients (h_WB.Rad_) obtained through experiments and simulation with the prior literature for a temperature difference (ΔT_surf-air_) of 9.5°C.

Considering our simulation results for the "manikin family", it is unlikely that inconsistencies in prior literature stem from differences in the body shapes of manikins utilized by different groups but come from varied methodologies. In particular, the presence of hair on the manikin’s head (scalp hair), variation in manikin surface temperature regulation (constant temperature mode versus heat flux mode), and the type of chamber walls (solid versus porous cloth wall), all could have compounding effects to the heat transfer coefficient variation. However, despite highly varied experimental procedures, Fig 8A shows relatively small differences in the total heat fluxes reported in prior literature and our work. Fig 8A, 8B show that most of the difference in the free convection heat flux estimation stems from how it was distinguished from radiative heat flux.

The convective heat transfer from a thermal manikin can be determined through several approaches accounting for radiative heat transfer, tabulated in S6 Table in S1 File, along with their limitations. These approaches include eliminating either the convective or radiative heat flux, direct flux measurement, and calculating or simulating the radiative flux followed by its subtraction from the total heat flux. Each approach has inherent shortcomings that can lead to inaccuracies in the reported heat transfer coefficients. For instance, when radiation is eliminated using low-emissivity aluminum foil [21, 24, 25] uneven spreading of the gelling agents or adhesives and air pockets between the foil and the manikin shell can cause spatial non-uniformity of exterior foil surface temperature. In addition, that temperature could be substantially different from the measured manikin shell temperature, resulting in skewed convective heat transfer coefficients. Also, the emissivity value for the aluminum foil has been assumed to have different values (e.g., 0.025 [25], 0.04 [48], and 0.1 [21]), whereas our FTIR measurements of several commercial tapes yielded 0.08 to 0.1. Likewise, the use of flux meters [48] or naphthalene discs attached to the manikin surface can also disrupt the temperature uniformity and alter the boundary layers, leading to underprediction of the heat transfer coefficients (as illustrated in Figs 5 and 7B in Kurazumi et al. [23]). Similarly, the h_WB.Rad_ values determined using equations recommended by the ASHRAE are typically 0.8 W·m^-2^°C^-1^ lower than our h_WB.Rad_ values (see Fig 7B and S9 Fig in S1 File). This discrepancy arises because ASHRAE’s h_WB.Rad_ expression assumes a view factor of 1 between the human body and the surrounding environment for a normal standing posture. In contrast, our manikin simulation yields a corresponding view factor of ~0.88 [37, 40, 44]. This difference propagates into overestimating the free convection coefficients (for example, for h_WB_ for ΔT_surf-air_ of 15°C employing the ASHRAE method yields 3.9 W·m^-2^°C^-1^ as compared to the 3.1 W·m^-2^°C^-1^ from our work).

### 4.2. Implication of our results on indoor overheating

Our current results on free convection combined with prior literature demonstrating that radiative [23] and forced convection [23] heat transfer coefficients are also nearly independent of body shape show that energy balance-based thermal stress and comfort calculations performed on a whole-body basis per surface area can be reliably applied across diverse body types. Therefore, for indoor thermal audits [23] or overheating risk assessments, using an average body shape to estimate heat exchange with the environment is sufficient. This conclusion is particularly relevant to estimations of indoor and outdoor conditions [49] that cause the human body to overheat. In particular, our work shows that the increased risks of heat illness associated with obesity [35, 36] are not caused by body shape affecting external heat transfer processes (e.g., a larger individual having higher heat flux) but rather by how heat is distributed within the body and the response of the thermoregulatory system to the internal heat. This insight demonstrates the general applicability of thermal audits and overheating risk predictions based on whole-body energy balance, aiding in designing more effective strategies for preventing heat stress in indoor environments.

### 4.3. Limitation of the current methodology

Delaminating the convective and radiative heat fluxes using coupled simulations with geometry approaching the experimental equivalent is the most realistic approach, but it still offers many opportunities for refinements. For instance, the model could replicate ANDI’s stand, connecting tubes and cables, measurement probes, and other miscellaneous experimental apparatus. Likewise, the model could be improved by incorporating unheated segments, including actuated joints (e.g., the armpit and crotch area) and fingers. Moreover, refining the assumptions about uniform air temperature outside the chamber and the overall heat transfer coefficient across the enclosure walls to account for the spatial variations can improve the model’s fidelity. With such improvements, an even more accurate representation of the physical setup can be simulated, leading to an improved prediction of the convection process. In future studies, clothing should be included to better reflect real-world conditions, as it acts as an insulating layer and significantly affects convective and radiative heat transfer, potentially impacting human thermal behavior. Future work should also explore other body postures, such as seated positions, where the type of chair can significantly influence heat transfer. In addition, other body shapes, including young children, elderly, pregnant women, or populations from other world regions, should be considered. These refinements and future directions will offer deeper insights into human thermal interactions, leading to more comprehensive and accurate conclusions about the heat transfer process.

## 5. Conclusions

Free convection plays a critical role in determining thermal comfort and safety in indoor environments. However, prior studies have primarily focused on average Western adults, leaving a significant gap in understanding how variations in body shape influence free convective heat transfer. This study addressed this gap by developing and validating a numerical model against experimental data from the thermal manikin ANDI. Experiments were conducted in a climate-controlled enclosure across air-to-skin temperature differences ranging from 4.9 to 13.9°C. We found overall differences of <5% between measured and simulated total heat fluxes across ANDI’s whole body and its anatomical regions, reaching at worst 15 to 20% locally for select (e.g., hands and torso) surface zones at the highest ΔT_surf-air_. Even these few more substantial disagreements are within typical uncertainties of convection measurements and likely stem from physical features, such as connecting cables in the lower back of ANDI or non-heated hands, that are not represented in the virtual ANDI model. The free convective heat transfer coefficients for ANDI that we determined by subtracting the simulated radiative flux from the total measured and simulated fluxes are within the wide range reported in prior literature (e.g., from 2.5 to 4.2 W·m^-2^°C^-1^ at ΔT_surf-air_ of 10°C). Furthermore, our regression coefficients and exponents can also be derived considering classical theory for free turbulent convection from a vertical plate. The flow being in the turbulent regime leads to the plate height canceling out in the heat transfer coefficient calculation, explaining why our whole-body heat transfer coefficient values are independent of the simulated manikins’ height (see Fig 7C). Regarding the large scatter in prior literature values, it likely stems from variation in experimental approaches and computation methods quantifying radiative heat exchange, not shape differences in the "average" manikins used by different groups.

Our simulations of convection around manikins in the "virtual family" representing 1^st^ to 99^th^ percentile BMI and height diversity of the U.S. adult population showed that body shape has a minor to negligible impact on this heat transfer process. Specifically, similar to findings from radiation and forced convective heat exchange studies [37, 38], variations in whole-body free convection coefficients remain below 3% for height and 8% for BMI, while regional variations across body shapes do not exceed 16%. Within this narrow range, our simulations show that the free convection heat transfer coefficient is nearly independent of the manikin height and sex but decreases proportionally to increase in the BMI percentile. Therefore, average body shape can be generally applied in whole-body energy balance-based thermal audits, thermal comfort (e.g., predicted mean vote), or overheating risk assessment models. The results and valid application of the models support critical insights for human health, productivity, and well-being connected to heat and cooling in buildings.

## Supporting information

S1 File NC supplemental information REVIEW 2. (PDF)
