# Supplementary material for: Impact of human body shape on free convection heat transfer
Source: PLoS One. 2025 Feb 6;20(2):e0318842. doi: 10.1371/journal.pone.0318842 (PMC11801630; doi:10.1371/journal.pone.0318842)
Supplement: S1 File — (PDF) [file pone.0318842.s001.pdf]

Supplemental Material for Impact of Human Body Shape on Free Convection Heat Transfer

Shri H. Viswanathan[1], Ankit Joshi,[1,2,] Lyle Bartels,[1] Kambiz Sadeghi,[1,2] Jennifer K. Vanos,[2,3] and Konrad Rykaczewski[1,2]*

1. *School for Engineering of Matter, Transport and Energy, Arizona State University, Tempe, AZ 85287, USA*
2. *Julie Ann Wrigley Global Futures Laboratory, Arizona State University, Tempe, AZ 85287, USA*
3. *School of Sustainability, Arizona State University, Tempe, AZ, USA*

Corresponding author: *konradr@asu.edu

1. **Schematic representation of the experimental setup and ANDI's shell**
2. **Effect of taping the gaps and actuated joints on ANDI surface on heat transfer measurements**
3. **Discrepancies in zonal surface areas between ANDI and its virtual twin**
4. **Empirically determining the overall heat transfer coefficient of the enclosure walls**
5. **Mesh Refinement Study**
6. **Transient behavior of local free convective coefficients and temperature contours**
7. **Geometry of the virtual room and segmentation of the average male manikin (50% BMI and height)**
8. **Re-segmentation of ANDI's 35 zones into 16 larger anatomical regions**
9. **Literature Study**
10. **Regression correlations for regional free convective heat transfer coefficients**
11. **Various delamination techniques and their limitations**
12. **Comparing the whole-body radiative coefficients computationally obtained in our work against other prior works**

1. Schematic representation of the experimental setup and ANDI's shell

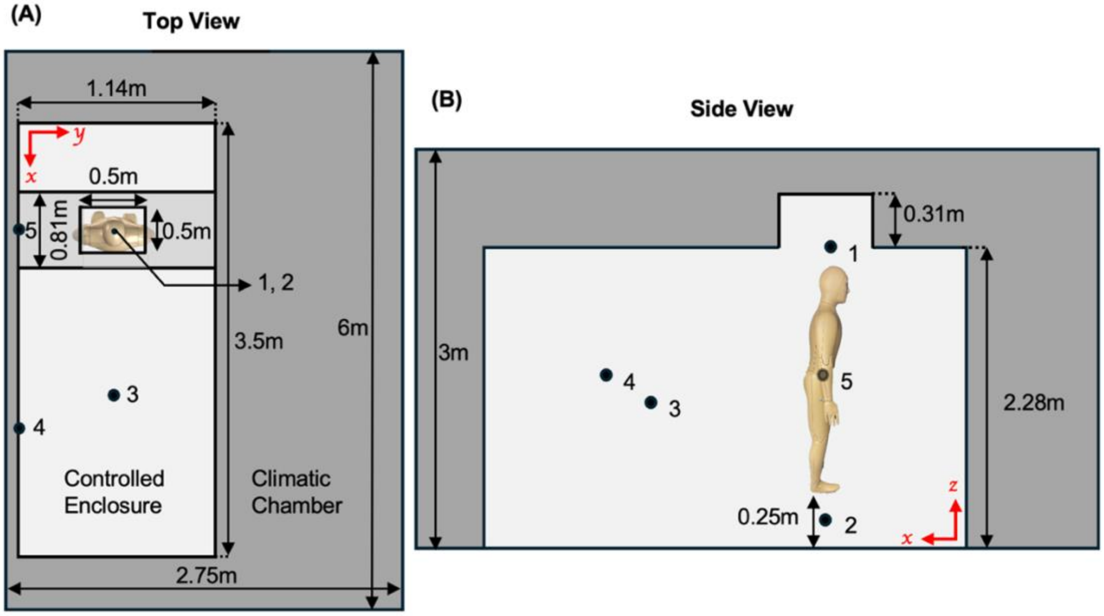

S1 Fig. Schematic representation of the experimental setup and ANDI's shell
(A) Top and (B) Side view of the schematic of the experimental setup illustrating the thermal manikin ANDI inside the controlled enclosure and the locations of the probes described in Table 1 and S1 Table.

S1 Table Coordinates of probe locations relative to the bottom left corner of the front face of the sealed enclosure (origin: X = 0, Y = 0, Z = 0).

Location	X (m)	Y (m)	Z (m)
1	1.27	0.57	2.21
2	1.27	0.57	0.12
3	2.62	0.57	1.06
4	1.35	0	1.16
5	2.69	0	1.16

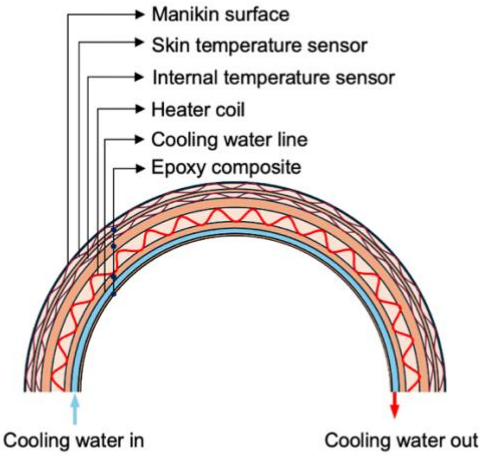

S2 Fig. Cross-sectional schematic of ANDI's shell

2. Effect of taping the gaps and actuated joints on ANDI surface on heat transfer measurements

While conducting the controlled experiments at five different air temperatures, we noticed that a part of the rising hot air plume escaping into the manikin's hollow shell through the adjustable joints such as armpits, elbows, waist, crotch, knees and ankles. Subsequently, the inner side of ANDI's shell (at 34 °C) also lost heat to the plume (at temperature < 34 °C). At whole-body level, ANDI lost 3 to 6.3% more heat when not taped compared to when taped. This effect was substantial (>5%), especially for front and rear torso, pelvis, calves, upper arms and forearms as depicted in Fig 1C. Thus, we closed the gaps created by the actuated joints using masking tape to prevent the air flow into the shell and accurately measure the heat exchange.

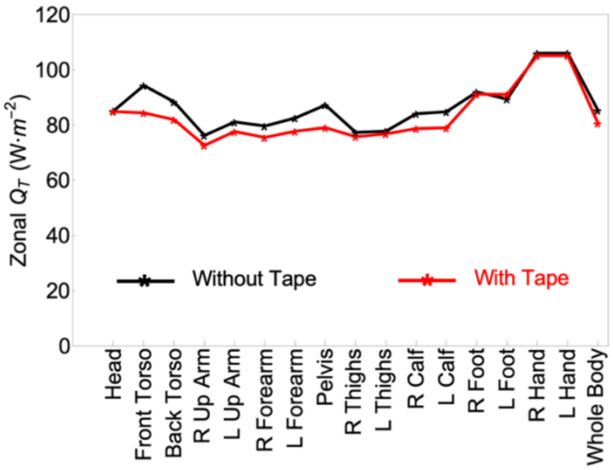

S3 Fig. Comparing the zonal total heat flux (Q_T) measured for a temperature difference of 9.5 °C with and without taping the actuated joints and gaps of ANDI.

3. Discrepancies in zonal surface areas between ANDI and its virtual twin

Substantial disagreement in surface areas occurs at segments closer to the actuated joints. These joints are either absent (upper arms) or smoothened (forearms) or have varying geometries (lower back, underarms and upper thighs). Further, the sharp edges present on the face segment surface was smoothened to avoid poor mesh quality. As for the hand region, the physical ANDI can only heat the inner and outer palms, and the fingers remain unheated. Moreover, creating boundary layers around the fingers produces low quality mesh elements. Hence, we created virtual hands without fingers. This geometrical difference led to the discrepancy in the surface area in hands.

S2 Table Discrepancies in zonal surface areas between ANDI and its virtual twin

Code	Zone	Virtual zonal area (m^2)	Actual zonal area (m^2)	% difference from Actual area	Code	Zone	Virtual zonal area (m^2)	Actual zonal area (m^2)	% difference from Actual area
1	Face	0.043	0.039	10.6	19	Left Underarm	0.033	0.031	7.8
2	Head	0.046	0.046	2.0	20	Upper Back	0.082	0.078	5.0
3	Neck	0.054	0.054	-0.9	21	Lower Back	0.077	0.058	31.8
4	Right Up Arm Front	0.063	0.053	18.3	22	Right Up. Thigh Front	0.062	0.068	-9.5
5	Right Up Arm Back	0.032	0.033	-2.2	23	Right Up. Thigh Back	0.042	0.043	-2.2
6	Left Up Arm Front	0.063	0.053	20.0	24	Left Up Thigh Front	0.062	0.068	-8.5
7	Left Up Arm Back	0.033	0.033	0.3	25	Left Up Thigh Back	0.042	0.043	-1.1
8	Right Forearm Front	0.043	0.045	-5.6	26	Right Low Thigh Front	0.105	0.105	0.0
9	Right Forearm Back	0.025	0.027	-8.2	27	Right Low Thigh Back	0.034	0.034	-0.8
10	Left Forearm Front	0.043	0.045	-5.0	28	Left Low Thigh Front	0.105	0.105	0.1
11	Left Forearm Back	0.025	0.027	-7.0	29	Left Low Thigh Back	0.034	0.034	-0.7
12	Right Hand	0.019	0.018	7.1	30	Right Calf Front	0.098	0.098	-0.2
13	Left hand	0.019	0.018	6.1	31	Right Calf Back	0.051	0.051	-0.2
14	Right Shoulder	0.016	0.017	-9.3	32	Left Calf Front	0.098	0.098	0.0
15	Left Shoulder	0.015	0.017	-13.3	33	Left Calf Back	0.051	0.051	-0.2
16	Chest	0.081	0.081	0.1	34	Right Foot	0.058	0.058	0.0
17	Stomach	0.080	0.077	4.3	35	Left Foot	0.058	0.058	0.2
18	Right Underarm	0.034	0.031	10.0	**Whole–Body**		**1.825**	**1.794**	1.7

4. Empirically determining the overall heat transfer coefficient of the enclosure walls

The wall temperatures measured in locations 4 and 5 were empirically matched to determine the overall heat transfer coefficient applied on the walls of the enclosure, with be 5.6 $W \cdot m^{-2} °C^{-1}$ found to be optimal.

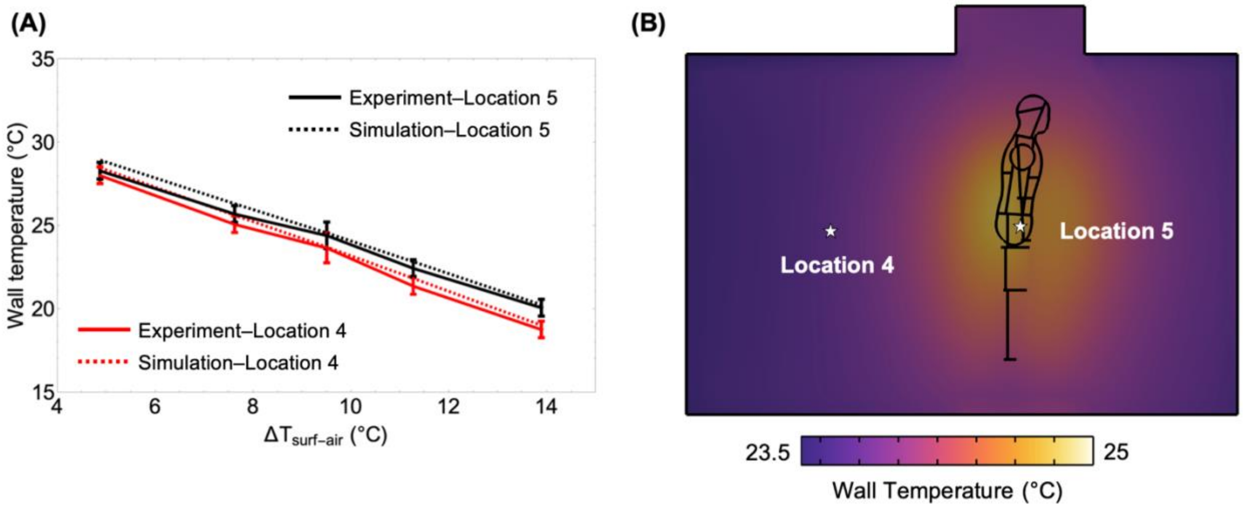

S4 Fig. Wall temperature characteristics

(A) Comparing the experimental and computational wall temperatures at locations 4 & 5 for temperature differences ($\Delta T_{surf\text{-}air}$) ranging between 4.9 to 13.9 °C and (B) Spatial non-uniformity of the wall temperature is illustrated computationally for $\Delta T_{surf\text{-}air}$ of 9.5 °C

5. Mesh Refinement Study

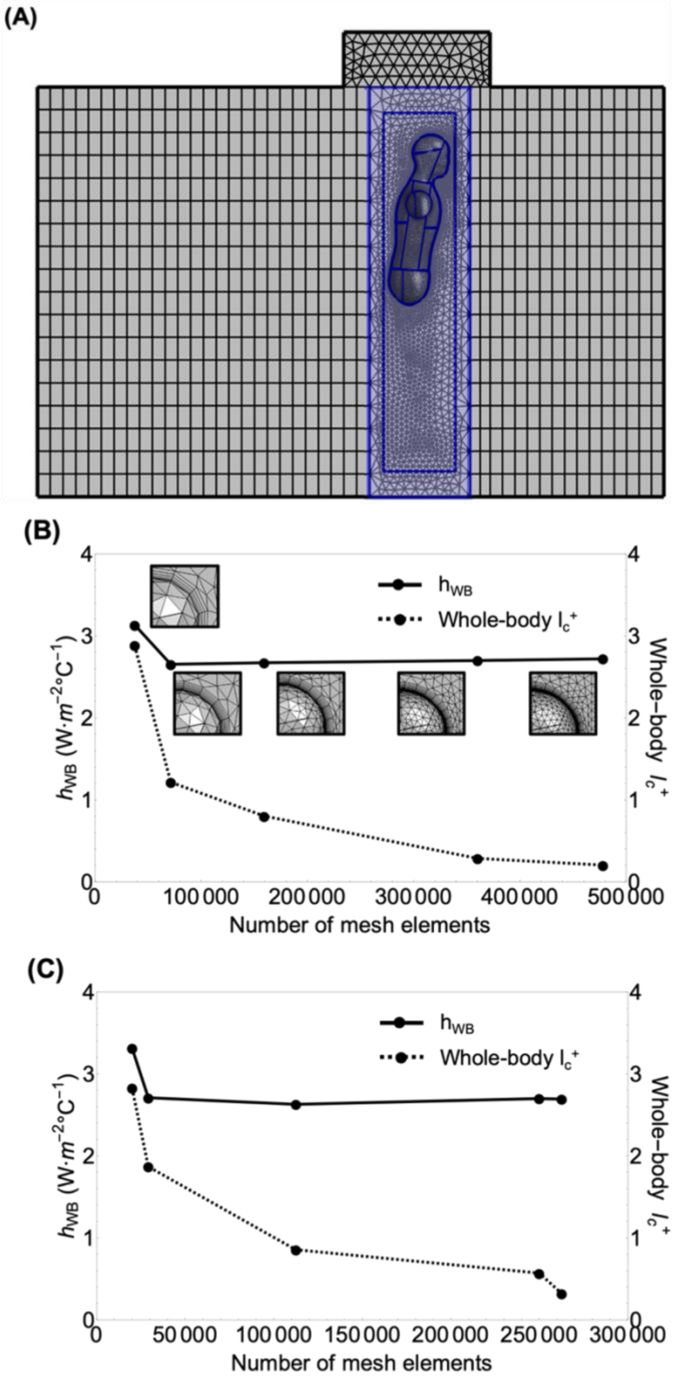

S5 Fig. Mesh refinement study

(A) Mesh schematic highlighting the two cuboidal refinement volumes, Mesh refinement study for illustrating the relationship between the number of mesh elements and the whole-body heat transfer coefficient as well as the whole-body dimensionless wall distance l_c^+ (B) for computationally replicating the experimental setup and (C) for simulating the family of "virtual manikins". Series of inset pictures in (B) illustrate the gradual refinement of the mesh elements at each step. l_c^+ is defined as the distance measured in viscous units, from the wall to the center of the wall adjacent cell.

6. Transient behavior of local free convective coefficients and temperature contours

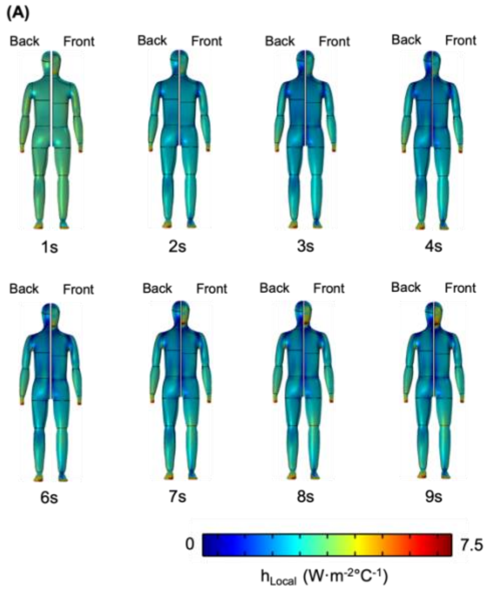

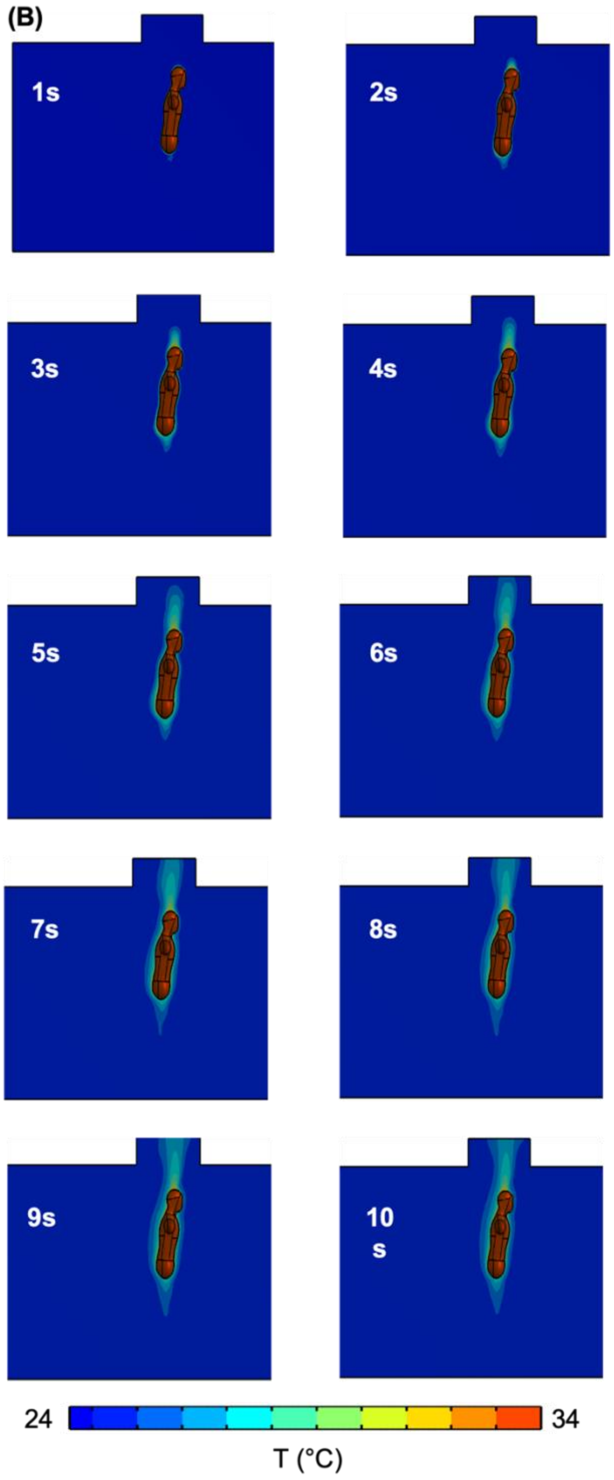

S6 Fig. Temporal evolution of the local free convective heat transfer coefficient and temperature contour

(A) Temporal evolution of the local free convective heat transfer coefficient distribution on the manikin surface over a 10-second interval and (B) Temporal evolution of the temperature contours over a 10-second interval.

7. Geometry of the virtual room and segmentation of the average male manikin (50% BMI and height)

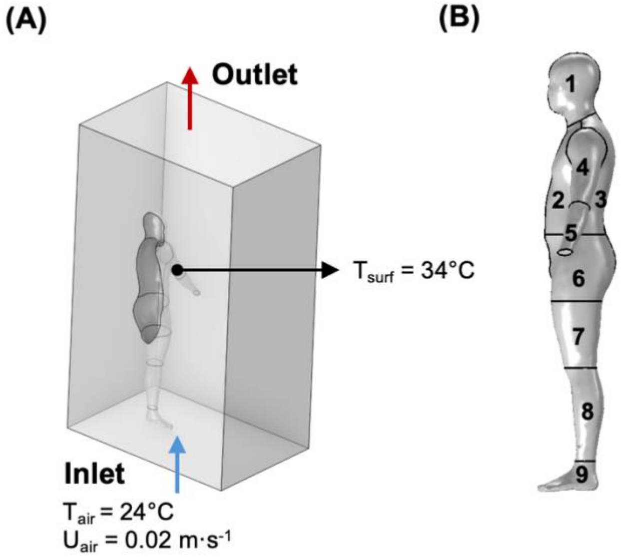

S7 Fig. Geometry of the virtual room and segmented average male manikin
(A) Schematic representation of the virtual room where T_{surf} – manikin surface temperature (°C), T_{air} – inlet air temperature (°C), U_{air} – inlet air speed (m·s^{-1}) and (B) depicts segmentation of the average male manikin (50% BMI and height).

8. Re-segmentation of ANDI's 35 zones into 16 larger anatomical regions

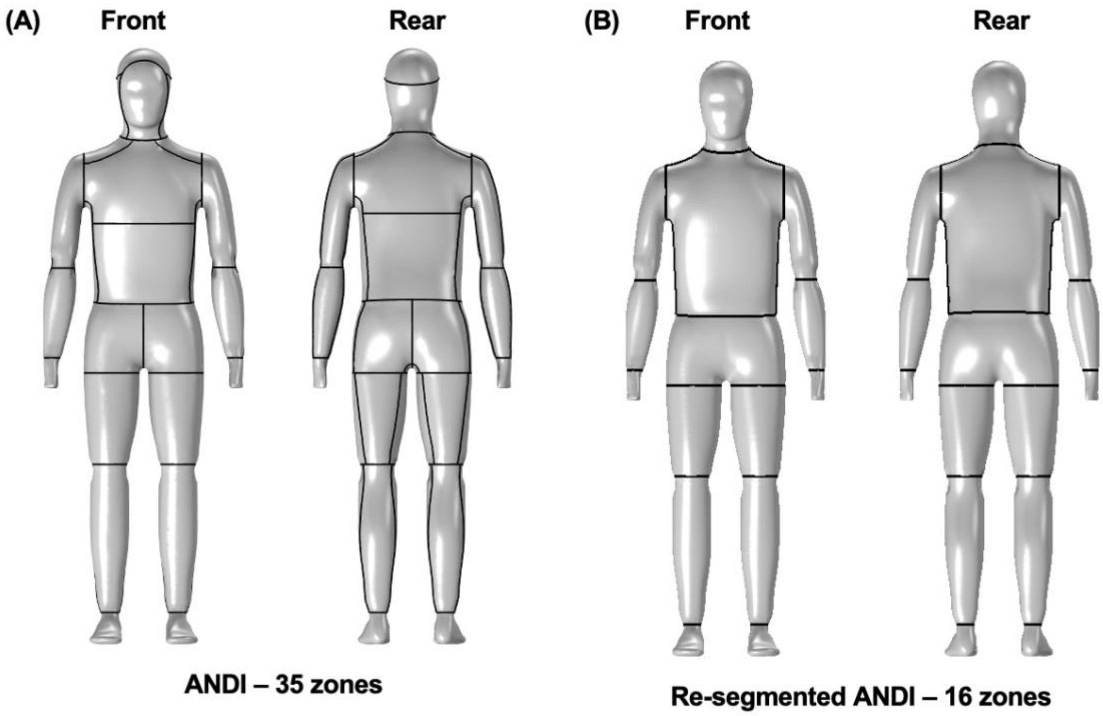

S8 Fig. Segmentation of ANDI's 35 zones into 16 larger anatomical regions

(A) Original segmentation of ANDI surface into 35 zones and (B) Simplified segmentation of ANDI surface into 16 zones.

S3 Table Re-segmentation of the original 35 zones ANDI into a simplified 16 zone configuration.

Re-segmented ANDI – 16 zones	ANDI – 35 zones
Head	Face (1), Head (2), Neck (3)
Front Torso	Chest (16), Stomach (17), half of [Right Shoulder (14), Left Shoulder (15), Right Underarm (18), Left Underarm (19)]
Back Torso	Upper Back (20), Lower Back (21), half of [Right Shoulder (14), Left Shoulder (15), Right Underarm (18), Left Underarm (19)]
R Up Arm	Right Up. Arm Front (4), Right Up. Arm Back (5)
L Up Arm	Left Up. Arm Front (6), Left Up. Arm Back (7)
R Forearm	Right Forearm Front (8), Right Forearm Back (9)
L Forearm	Left Forearm Front (10), Left Forearm Back (11)
R Hand	Right Hand (12)
L Hand	Left Hand (13)
Pelvis	Right Up. Thigh Front (22), Right Up. Thigh Back (23), Left Up. Thigh Front (24), Left Up. Thigh Back (25)
R Thigh	Right Low Thigh Front (26), Right Low Thigh Back (27)
L Thigh	Left Low Thigh Front (28), Left Low Thigh Back (29)
R Calf	Right Calf Front (30), Right Calf Back (31)
L Calf	Left Calf Front (32), Left Calf Back (33)
R Foot	Right Foot (34)
L Foot	Left Foot (35)

9. Literature Study

S4 Table Literature Study

Reference	Operation Mode	Air temperature Tair (°C)	Mean Radiant Temperature MRT (°C)	Skin Temperature (°C)	Air Velocity ($m \cdot s^{-1}$)	Manikin emissivity	Whole-body free convective coefficient ($W \cdot m^{-2} °C^{-1}$)	Radiative coefficient ($W \cdot m^{-2} °C^{-1}$)
de Dear et al.[5]	Skin temperature control	Not mentioned	T_{air} = MRT	Not mentioned	< 0.1	0.95	3.4	4.5
Oliveira et al. [11]	Thermal comfort mode	12.9–13.7	T_{air} - $T_{operative}$ < 0.2	$36.4 - 0.054Q$	Not mentioned	0.95	3.5	4.58
Kurazumi et al. [9]	Skin temperature control	16–26	T_{air} = MRT	33	< 0.2	0.98	$1.007(\Delta T_{surf\text{-}air})^{0.406}$	4.432
Quintela et al. [4]	Heat flux control	13–29	T_{wall} = 12–30	$36.4 - 0.054Q$	Not mentioned	0.95	$2.02(\Delta T_{surf\text{-}air})^{0.24}$	4.58
Kilic et al.[2]	Heat flux control	22	27–29	$32.6 + 0.066T_{air} + 32W_a$	0.14	0.98	3.76	4.59
Oguro et al. [10]	Skin temperature control	15	Not mentioned	20–36.5	< 0.1	Not mentioned	$1.21(\Delta T_{surf\text{-}air})^{0.43}$	Not mentioned
Fojtlin et al. [8]	Skin temperature control	24	T_{air} = MRT	34	0.05	0.95	3.77	4.4 –4.6

10. Regression correlations for regional free convective heat transfer coefficients

S5 Table Regression correlations for regional free convective heat transfer coefficients

Coefficients and exponents of the nonlinear regression power function of $h_{Local} = A \cdot (\Delta T_{surf\text{-}air})^{B}$ with 90% confidence interval values based on the simulation of ANDI inside the experimental setup. The input temperature difference is in degrees Celsius. We assume the entire geometry to be symmetrical across the sagittal plane.

Zones	A	B
Head	1.02±0.003	0.45±0.001
Front Torso	0.91±0.028	0.40±0.014
Back Torso	0.85±0.005	0.40±0.003
Upper Arm	1.39±0.007	0.28±0.002
Forearm	1.83±0.023	0.24±0.005
Pelvis	1.06±0.01	0.36±0.004
Thighs	1.36±0.022	0.30±0.007
Calf	1.61±0.013	0.25±0.003
Foot	2.46±0.012	0.21±0.002

11. Various delamination techniques and their limitations

S6 Table Techniques adopted in the other prior works to delaminate convection from radiation

Technique	Procedure	Limitation	Reference
**Convection Elimination** $C \cong 0$ $Q_T \cong R$	Maintain $T_{surf} = T_a \neq T_{MRT}$	-	Ichihara et al. [1], Joshi et al. [6]
**Radiation Elimination** $R \cong 0$ $Q_T \cong C$	Heat flux meters wrapped with Al foil	Flux meters interfere with the boundary layer formation around the manikin surface	Danielsson et al. [7]
	Manikin surface covered with Al foil	Uneven spreading of gelling agents leads to non-uniform surface temperatures and measurement errors	Quintela et al. [4], de Dear et al.[5], Fojtlin et al. [8]
**Direct Measurement**	C measured through naphthalene sublimation	Preparing naphthalene discs is time and labor-intensive process	
	R measured using radiant flux meters	Sensors disturb the temperature uniformity and boundary layer formation	Kurazumi et al. [9]
**Calculation**	h_r computed from ASHRAE Handbook $h_r = 4\epsilon\sigma(A_r/A_D)[273.2 + (T_{cl} + T_{MRT}/2]^3$	View factor between skin and environment is ignored	Oguro et al. [10], Oliveira et al. [11]
**Simulation**	C and R are numerically solved, using CFD models with realistic 3D human geometries	-	Kilic et al.[2]

$$Q_T = C + R = h_c(T_{sk} - T_{air}) + h_r(T_{sk} - MRT) \qquad \text{Eq.1}$$

Q_T – Total heat flux ($W \cdot m^{-2}$)
C – Convective heat flux ($W \cdot m^{-2}$)
R – Radiative heat flux ($W \cdot m^{-2}$)
h_c – Convective heat transfer coefficient ($W \cdot m^{-2} {}^{\circ}C^{-1}$)
h_r – Radiative heat transfer coefficient ($W \cdot m^{-2} {}^{\circ}C^{-1}$)
T_{sk} – Skin temperature (°C)
T_{air} – Air temperature (°C)
MRT – Mean Radiant Temperature (°C)

12. Comparing the whole-body radiative heat transfer coefficients of our work obtained computationally with ANDI against prior works

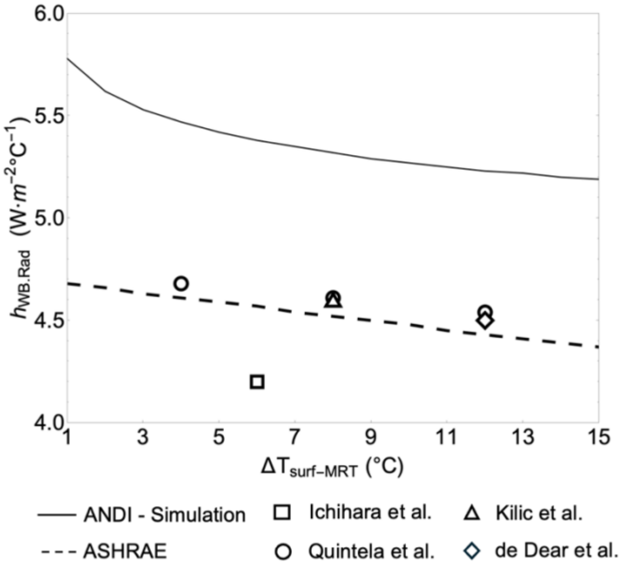

S9 Fig. Comparison of whole-body radiative heat transfer coefficients ($h_{WB.Rad}$) obtained from simulation with ANDI against prior literature (Ichihara et al. [1] , Kilic et al.[2], ASHRAE [3], Quintela et al. [4], and de Dear et al.[5])for temperature difference between the manikin surface and mean radiant temperature ($\Delta T_{surf\text{-}MRT}$) ranging between 1 to 15 °C.

References

[1] M. Ichihara, Measurement of Convective and Radiative Hear Transfer Coefficients for the Standing and Sitting Human Body Using a Thermal Manikin, J. Archit. Plann. Environ. Eng., AIJ 501 (1997) 45–51.

[2] M. Kilic, G. Sevilgen, Modelling airflow, heat transfer and moisture transport around a standing human body by computational fluid dynamics, International Communications in Heat and Mass Transfer 35 (2008) 1159–1164.

[3] ASHRAE, Handbook of Fundamentals, American Society of Heating, Refrigerating and Air-Conditioning Engineers Inc., Atlanta, 2001.

[4] D. Quintela, A. Gaspar, C. Borges, Analysis of sensible heat exchanges from a thermal manikin, Eur J Appl Physiol 92 (2004) 663–668.

[5] R.J. De Dear, E. Arens, Z. Hui, M. Oguro, Convective and radiative heat transfer coefficients for individual human body segments, Int J Biometeorol 40 (1997) 141–156.

[6] A. Joshi, S.H. Viswanathan, A.K. Jaiswal, K. Sadeghi, L. Bartels, R.M. Jain, G. Pathikonda, J.K. Vanos, A. Middel, K. Rykaczewski, Characterization of human extreme heat exposure using an outdoor thermal manikin, Science of the Total Environment 923 (2024) 171525.

[7] U. Danielsson, Convection coefficients in clothing air layers., (1996).

[8] M. Fojtlín, J. Fišer, M. Jícha, Determination of convective and radiative heat transfer coefficients using 34-zones thermal manikin: Uncertainty and reproducibility evaluation, Exp Therm Fluid Sci 77 (2016) 257–264.

[9] Y. Kurazumi, T. Tsuchikawa, J. Ishii, K. Fukagawa, Y. Yamato, N. Matsubara, Radiative and convective heat transfer coefficients of the human body in natural convection, Build Environ 43 (2008) 2142–2153.
[10] M. Oguro, E. Arens, H. Zhang, T. Katayama, Convective heat transfer coefficients and clothing insulations for parts of the clothed human body under airflow conditions, Journal of Architecture and Planning (Transactions of AIJ) 67 (2002) 21–29.
[11] A.V.M. Oliveira, A.R. Gaspar, S.C. Francisco, D.A. Quintela, Analysis of natural and forced convection heat losses from a thermal manikin: Comparative assessment of the static and dynamic postures, Journal of Wind Engineering and Industrial Aerodynamics 132 (2014) 66–76.
